# Monthly out-of-pocket caps substantially increase actuarial value for high-deductible health plans: a simulation study

**DOI:** 10.1093/haschl/qxag122

**Published:** 2026-05-21

**Authors:** David M Anderson, Paul R Shafer

**Affiliations:** Department of Health Services Policy and Management, University of South Carolina, Columbia, SC 29208, United States; Department of Health Law, Policy and Management, Boston University, Boston, MA 02118, United States

**Keywords:** Affordable Care Act, health insurance, policy analysis, Centers for Medicare and Medicaid Services, catastrophic plans, marketplaces

## Introduction

The affordability of health insurance and health care has remained a pressing national concern despite progress made under the Affordable Care Act (ACA), amplified by expiration of enhanced premium tax credits at the end of 2025. Underinsurance is a substantial problem, where patients cannot afford to pay large amounts before their coverage kicks in, with individuals who are not eligible for premium subsidies facing both high premium and out-of-pocket (OOP) costs. Catastrophic plans in the ACA Marketplace, previously only available to those under age 30 years or with a hardship exemption, are available to anyone with an income above 250% of the federal poverty level starting in 2026. Catastrophic plans have lower premiums for nonsubsidized individuals than Bronze plans but, for 2027, both tiers of plans have annual deductibles equal to the annual OOP maximum as a standardized feature, capped at $12 000 for self-only coverage.^1^

Large annual deductibles serve as a barrier to health care use; yet, decades have passed with little innovation to this design feature of commercial plans. Currently, Medicare Part D beneficiaries may spread the payment of the annual deductible over the year to better manage liquidity. Monthly cost-sharing limits may reduce financial burdens in theory, but have yet to be implemented.^2^ In the 2027 proposed rule for the Marketplace, the Centers for Medicare and Medicaid Services (CMS) proposed allowing catastrophic plans to have monthly cost-sharing limits equal to one-twelfth of the maximum annual deductible.^1^

Little is known about the potential changes in actuarial value, or conversely, the costs borne by insurers, with monthly cost-sharing limits. In this simulation study, we assessed the potential impact of the proposed policy change by simulating actuarial value over a range of hypothetical annual and monthly cost-sharing limits.

## Methods

We used the 2023 Merative™ MarketScan® Commercial Claims and Encounters, one of the largest sources of national claims data for commercial plans. We limited the sample to those under age 65 with full-year coverage, not in a Health Maintenance Organization (HMO) or point of service (POS) with capitation plan, and in a high-deductible health plan (*n* = 3 326 028), our best-available proxy of catastrophic plan enrollment. Claims were restricted to in-network services and we dropped the most expensive half-percent of patients, similar to CMS practice in defining actuarial value.

We first calculated actuarial value as the quotient of one minus annual OOP costs divided by the total allowed amount (expenditures after applying the insurer-negotiated rate) for the year, estimating baseline actuarial value in our sample. We then simulated multiple hypothetical scenarios, reprojecting OOP cost burdens and actuarial value based on the observed claims at the enrollee level. These scenarios included a (1) $12 000 annual OOP maximum (the maximum allowed for 2027 per CMS), (2) $1000, (3) $2000, (4) $3000, (5) $4000, and (6) $5000 monthly OOP maximum. These simulated scenarios did not include separate deductibles, coinsurance, or copayments; rather, all claims were only subjected to a monthly OOP maximum after which everything was covered at 100%, similar to the structure of the catastrophic plans to which the proposed policy would apply.

We acknowledge several limitations. First, nominal spending in 2023 was lower than 2027 will be given inflation; however, the relative difference in actuarial value should remain constant. Second, we cannot account for Health Savings Account balances or employer contributions, although the latter are not relevant for the Marketplace. Third, we cannot account for behavioral response to cost-sharing changes that may alter demand for care. However, we estimate plausible alternative behavioral responses using CMS estimates of induced demand factors, applying Silver to Gold Bronze to Gold adjustments, and another based on Brot-Goldberg et al,^3^ inverting their result and finding a 42% reduction in spending when under the deductible.

Our analyses were conducted in SAS version 9.4 and Stata/MP version 19. The Boston University Medical Campus Institutional Review Board deemed this study to be exempt.

## Results

Average baseline actuarial value and hypothetical $12 000 annual in-network OOP maximum were virtually identical at 78.9% (Table 1). A $1000, $2000, and $3000 monthly OOP maximum would increase actuarial value by 6.8% (to 84.2%), 3.0% (to 81.2%), and 1.3% (to 79.9%), respectively. A $4000 and $5000 monthly OOP maximum were within one-half percent of the baseline actuarial value at 79.3% and 79.0%, respectively. Our alternative analyses incorporating potential behavioral response increased actuarial value by smaller amounts using the CMS estimates and by approximately 4–5 percentage points using the Brot-Goldberg et al estimates.

**Table 1:** Simulated actuarial value under hypothetical cost-sharing scenarios among high-deductible health plan enrollees, 2023.

Scenario	Patient cost-sharing, %	Actuarial value, %	Actuarial value relative to status quo, %	Actuarial value with behavioral response (CMS, 2025; Silver to Gold), %	Actuarial value with behavioral response (CMS, 2025; Bronze to Gold), %	Actuarial value with behavioral response (Brot-Goldberg et al, 2017), %
Baseline (status quo)	21.1	78.9	—	—	—	—
$12 000 annual limit	21.1	78.9	100.1	—	—	—
$1000 monthly limit	15.8	84.2	106.8	84.8	85.2	88.3
$2000 monthly limit	18.8	81.2	103.0	81.9	82.4	85.8
$3000 monthly limit	20.1	79.9	101.3	80.6	81.1	84.7
$4000 monthly limit	20.7	79.3	100.6	80.1	80.5	84.1
$5000 monthly limit	21.0	79.0	100.2	79.8	80.3	83.9

The estimated actuarial values based on behavioral responses adjustments are in order of increasing size, from left to right. The first 2, based on CMS (2025), apply a 1.0485 (Silver to Gold) and 1.08 (Bronze to Gold) multiplier, respectively, to plan spending for all member-months. The third, based on Brot-Goldberg et al,^3^ applies a 1.724 multiplier (equal to 1/0.58) to plan spending only for member-months where the respective monthly limit was reached, inverting their result finding a 42% reduction in spending when under the deductible.

Abbreviation: CMS, Centers for Medicare and Medicaid Services.

## Discussion

In this simulation study of cost-sharing and actuarial value under varying monthly in-network OOP maximums, we found that simply dividing annual values by 12 yielded increased actuarial value. Actuarially equivalent policies would require a substantially higher monthly OOP maximum than this simple ratio.

These results build upon prior research finding that most patients do not have persistent interaction with the health care system; instead, spending is temporally clustered.^4^ Individuals could have many months without medical spending but then experience a single expensive medical event. For example, under current benefit design, 2 individuals with $12 000 in annual expenditures face the same cost-sharing regardless of timing, but with monthly cost-sharing limits, the distribution of spending can lead to highly variable cost-sharing. A patient with a single $12 000 event, such as a noncomplicated delivery, would only bear the monthly OOP maximum in 1 month, with the insurer covering the rest, while a patient with a monthly prescription would still face that cost every month. As such, monthly cost-sharing limits may benefit individuals with sporadic health care costs more. However, if CMS chose to make them actuarially equivalent, then an annual aggregate cap on OOP costs would still be warranted to protect patients against facing recurring cost burdens.

Higher actuarial value plans will, all else being equal, increase monthly premiums. However, enrollees in comparatively good health with low expected health care may highly value low premiums. Catastrophic plans are, for nonsubsidized enrollees, the least expensive plans on the Marketplace,^5^ but higher premiums may encourage potential enrollees to go without coverage. Monthly cost-sharing limits have potential to ease the burden of underinsurance^5^; however, catastrophic Marketplace plans may not be the optimal setting for their deployment given the price-sensitive population they serve.

## Supplementary Material

qxag122_Supplementary_Data

## Data Availability

Data is available for purchase as the Merative™ MarketScan® Commercial Claims and Encounters database. All data analysis was performed under a data use agreement.
